# Biological Characteristics of Captive Chinese Wuzhishan Minipigs (*Sus scrofa*)

**DOI:** 10.1155/2014/761257

**Published:** 2014-08-20

**Authors:** Fangui Min, Jinchun Pan, Xilong Wang, Rui'ai Chen, Fengguo Wang, Shuming Luo, Jiancong Ye

**Affiliations:** ^1^Guangdong Provincial Key Laboratory of Laboratory Animals, Guangdong Laboratory Animals Monitoring Institute, Guangzhou 510663, China; ^2^Guangdong Dahuanong Animal Health Products Stock Co., Ltd., Xinxing 527439, China

## Abstract

In order to meet the demands of experimental minipigs for biomedical researches, we have aimed at cultivating grazing Chinese Wuzhishan (WZS) minipigs and trying to make them useful and affordable since the 1990s. After more than ten years of captive cultivation following sound management practices and a rigorous selection program for fertility and litter size, we established an outbred WZS minipigs colony with a core group (14 males and 30 females) and an expanding group (20 males and 40 females). In 2010–2013 periods, extensive background data of this colony were recorded and analyzed. This paper was written to provide pertinent information about outbred WZS minipigs for producers, users, and others concerned with WZS minipigs. It contains physical characteristics, growth performance, productive performance, hematology and blood biochemistry, microsatellite analysis, organ coefficients, and carcass properties. Results show that WZS minipigs have characteristics of small body size, slow growth rate, long life cycle, high reproductive rate, and maintaining original genetic diversity. All data present that outbred WZS minipigs are suitable laboratory animal and model animal.

## 1. Introduction

Minipigs, or sometimes potbellied pigs, are strains of domestic pigs that are markedly smaller than farmyard varieties. For their low economic traits, the selection and cultivation of minipigs were far behind domestic farm pigs. Because of anatomical and functional similarities to humans or because of availability of disease models, pigs begin to be used as research models in the field of skin, cardiovascular system, urinary system, and metabolic syndrome [1–3], while the minipigs show a significant advantage over the domestic farm pigs for the reduced size which reduces the compound needs consequential prohibitive costs for the experiments and makes animals handling easier.

The cultivation of miniature pigs began in the 1940s, and more than 10 strains had been used in biomedical researches till the 1980s [4]. In Europe, the Göttingen minipig is the most popular breed used by pharmaceutical companies and contract research organizations. In the USA, strains of Yucatan mini- and micropigs, Sinclair minipigs, Minnesota Hormel minipigs, and Hanford minipigs are widely used today. Though these minipig strains are known as multispecies hybrid bred, their background information is clear and their hereditary stabilities are satisfied. China has rich resources of minipigs such as Chinese Wuzhishan (WZS) minipigs (*Sus scrofa*), Guizhou minipigs, Bama minipigs, and Tibet minipigs, but the laboratory animal work is far behind the developed countries. Compared with the strains used in developed countries, the Chinese origin minipigs are formed naturally with high genetic homozygosity and stable phenotype [5], indicating that Chinese origin minipigs are more ideal as laboratory animals for biomedical researches.

WZS minipig is a special pig in China initially grazing in isolated tropical areas in Hainan. It was firstly found and preserved in the 1980s; after that, the laboratory WZS minipigs began to be cultivated [6] and used in biomedical researches [7, 8]. Nowadays, one WZS minipigs colony for 20 generations of inbreeding has been established with a high inbreeding coefficient (more than 0.965) [9, 10]. Here we will introduce the outbred WZS minipigs that have been cultivated since the 1990s. After more than ten years of phenotypic selection, downsizing cultivation, and microbiological standardization, we established an outbred WZS minipigs colony and highlighted its background data for its usefulness in modern laboratory animal science, especially for being suitable for long-term studies in which domestic swine breeds become too large.

## 2. Material and Methods

### 2.1. Experimental Animals

An outbred WZS minipigs colony with a core group (14 males and 30 females) and an expanding group (20 males and 40 females) was set as the objective. All data were collected from them and their offspring in 2010–2013 periods. The administrative license number is* SCXK (Yue)2008-0022*.

Animals were bred in closed shelter with cooling water curtains and air supply fans in summer and infrared thermal equipment in winter. Environmental temperatures were normally kept in 18–29°C and air exchanges were maintained at about 10 times per hour.

### 2.2. Clinical Observation

Animals were observed by animal care technicians daily and veterinarian biweekly. All clinical signs were recorded daily and verified by veterinarian periodically.

### 2.3. Determination of Body Weight and Body Measurement

Body weight (BW) and body measurements were determined monthly up to 12 months and then at the interval of 6 months for BW. BW was taken by weighbridge and body measurements were made using the tailor's tape measure.

Height-at-withers (HAW) was measured as the straight-line distance from the withers to the base of the foot in a normal upright manner. Body length (BL) was measured as the distance from scapula to tail. Heart girth (HG), heart depth (HD), and heart breadth (HB) represented the circumference, straight-line distance, and horizontal distance of the chest posterior to the scapula, respectively. Abdomen girth (AG) showed the circumference of abdomen. Shank girth (SG) showed the circumference of the lower parts of the foreleg. Buttock-knee length (BKL) was measured from the most posterior point on either buttock to the anterior point of the knee. Buttocks breadth (BB) was measured as the horizontal distance between buttocks. Head length (HL) was measured from the distalmost dorsal point of the rhinarial pad to parietal. Forehead breadth (FB) was measured as the distance between the eyes. Jaw width (JW) was the distance between two mandibular angles. Caudal length (CL) was the distance from tail root to tail tip. Caudal girth (CG) was the circumference of tail root.

### 2.4. Measures of Reproductive Performance

The reproductive performances of the colony were monitored continuously for at least 3 birth frequencies according to* Records for Swine Breeds (GB 3038-82)*, Chinese national standards. Reproductive behavior parameters include male first penis protruding time, male first ejaculation time, female puberty, and female gestation. Litter performances include litter sizes, litter weights, and individual weights at birth, 21 d, and weaning (45 d).

### 2.5. Haematology and Blood Biochemistry

Nineteen haematological indices were determined by automatic hematology analyzer (Sysmex XT-2000iv), and 12 blood biochemistry indices were determined by automatic biochemical analyzer (Hitachi 7020).

### 2.6. Microsatellite Analysis

Twenty-six commonly used microsatellite loci were employed to analyze the genetic diversity of the outbred WZS minipigs by multi-PCR as previous report [12]. The loci and grouping in multi-PCR were listed in Table 1. Samples of ears or blood from 44 randomly selected animals were used.

### 2.7. Organ Coefficients and Carcass Properties

The minipigs (females and castrated males) were slaughtered according to standard procedures. The internal organs were removed and weighed as described in rats [13]. After that, the organ coefficients were counted, which were relative (% of body weight) organ weights. Then the carcass properties were determined according to* Chinese Technical Regulation for Testing of Carcass Traits in Lean-Type Pigs (NY/T825-2004)* [14].

### 2.8. Data Analysis

Data are expressed as means ± SD. Student's* t*-tests were used to analyze between-group differences. Variances of different time points in the same group were analyzed by one-way ANOVA. A value of* P* less than 0.05 was considered statistically significant. All analyses were done by using SAS software, version 8.01 (SAS Institute, Cary, North Carolina).

## 3. Results and Discussion

### 3.1. Physical Characteristics

**Figure 1:**
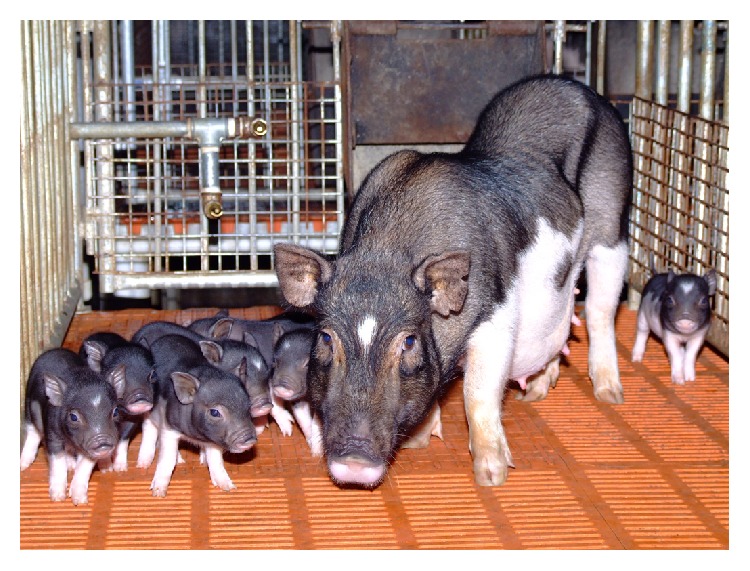
Morphological appearance of outbred WZS minipig.

Figure 1 shows the typical characteristics of outbred WZS minipigs. WZS minipigs have low body weight, slim body shape, and a small head with a long snout. Each trotter (foot) has four hoofed toes with the two larger central toes bearing most of the weight. The dental formula of adult pigs is the same as domestic pigs, giving a total of 44 teeth. The most common appearance of WZS minipigs is a black or brown body with white abdomen and legs and a white triangle on its forehead. Original WZS minipigs are normally active with sensitive response, while the temperament of outbred WZS minipigs tends to be gentle as domestic pigs. Detailed data on temperament and behaviour of outbred WZS minipigs will be evaluated and compared with original and inbred WZS minipigs in future studies.

### 3.2. Growth Performance

#### 3.2.1. Body Weight

**Figure 2:**
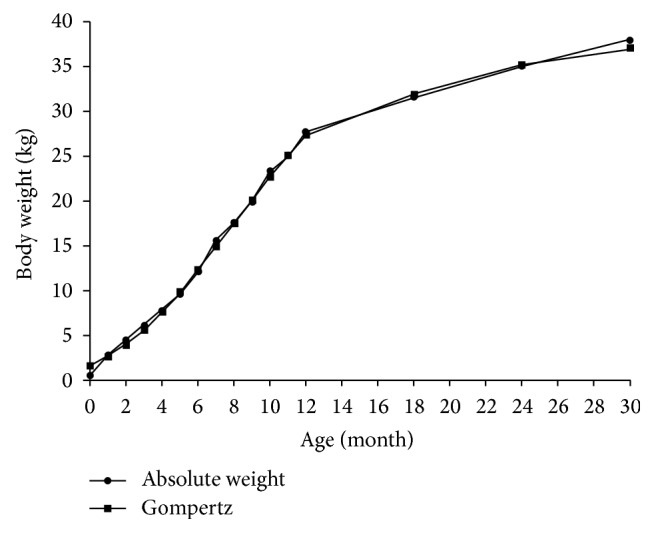
Growth curve for body weights of outbred WZS minipigs.

The minipigs were weighed routinely at a month interval up to 12 months and then at the interval of 6 months. All data are shown in Table 2. The body weights resemble Göttingen minipigs, the famous and widely used minipigs [15, 16]. The mean body weights of birth, 2 months old, 6 months old, and 12 months old are as follows: 0.57 ± 0.11 kg, 4.54 ± 1.66 kg, 12.11 ± 2.99 kg, and 26.21 ± 6.24 kg. The mean body weight of 6 months old is higher than that of WZS minipigs bred in original region (8.41 ± 2.13 kg) [17], but it is lower than that of WZS minipigs bred in Beijing, nonoriginal region (13.43 ± 3.27 kg) [18]. It is also lower than other Chinese famous minipigs such as Guizhou minipigs [19] and Banna mini- and micropigs [19]. In Figure 2, as age increases, the body weights show a rapid increase at the first year (12 months) and then increase slightly. Body weight data were further analyzed by the following nonlinear theoretical models as described previously [16]: logistic, Gompertz, Bertalanffy, and Richards. Though all the* R* ^*2*^ of nonlinear theoretical models are above 0.99, the Gompertz growth model is more suitable for our research for the maximum body weight drawing from Gompertz growth model is more practical (Figure 2). According to the Gompertz growth model, the maximum weight, inflection point of age, inflection point of body weight, and maximum daily increase of body weight are 45.82 kg, 230.11 d, 16.86 kg, and 0.0883 kg, respectively.

#### 3.2.2. Body Measurement

Fifteen indices of body measurements were carried out monthly up to 12 months and results were shown in Table 2. The mean values of body height, body length, chest depth, chest breadth, chest circumference, abdomen circumference, shank circumference, buttocks length, buttocks breadth, head length, forehead breadth, jaw width, caudal length, and caudal circumference of six-month-old WZS minipigs are as follows: 32.64 ± 3.81 cm, 52.13 ± 5.43 cm, 16.80 ± 2.09 cm, 13.95 ± 1.61 cm, 50.64 ± 5.77 cm, 58.30 ± 5.88 cm, 8.51 ± 0.74 cm, 18.77 ± 2.29 cm, 11.95 ± 1.31 cm, 17.75 ± 1.47 cm, 7.93 ± 0.54 cm, 9.74 ± 0.90 cm, 14.90 ± 2.40 cm, and 5.10 ± 0.68 cm. These data are a little higher than the values of WZS minipigs bred in original region and lower than the values of WZS minipigs bred in Beijing. And they are also lower than Chinese Guizhou minipigs [19] and Banna mini- and micropigs [19]. The mean body measurements values of 12-month-old WZS minipigs are as follows: 42.96 ± 3.37 cm, 67.38 ± 6.35 cm, 22.94 ± 2.82 cm, 17.59 ± 2.74 cm, 67.49 ± 7.74 cm, 74.69 ± 9.19 cm, 10.63 ± 0.78 cm, 24.75 ± 2.28 cm, 15.86 ± 1.64 cm, 23.19 ± 1.58 cm, 9.68 ± 0.70 cm, 12.70 ± 1.17 cm, 19.13 ± 2.11 cm, and 6.72 ± 0.72 cm.

The body measurements changing curves are the same as that of body weight showing rapid to slight increase. But the increases of body measurements in 1st month are significantly sharper than other months. Results show that WZS minipigs have the biological characteristics of small size and slow growing.

### 3.3. Reproductive Performance

#### 3.3.1. Reproductive Behavior Parameters

The sexual mature time of males is earlier than that of females in WZS minipigs. For males, the first penis protruding time is 39.56 ± 2.35 d (*n* = 45), and the first ejaculation time is 61.29 ± 3.07 d (*n* = 36). For females, puberty, the age of females emerging first estrus, is 93.65 ± 3.50 d. Females in estrus will often assume the lordosis reflex, and the vulvar lips are swollen and red with a thin, mucous discharge. They also show some other signs of estrus including depressed appetite, restlessness, alertness, pacing, grunting, and chomping of the jaws. The period from the last end of estrus to this end, called estrus cycle, is 21.16 ± 0.75 d for female WZS minipigs. And the behavioral estrus sustains 4.18 ± 0.31 d. After fertilization, females will experience 113.0 ± 2.33 d gestation till parturition.  Table 3 describes the main reproductive behavior parameters of female WZS minipigs.

#### 3.3.2. Litter Performance

The overall litter characteristics of the present study are given in Tables 4 and 5, and the differences in litter performances are also shown in them. In Table 4, the effects on litter sizes caused by birth frequencies are illustrated. As birth frequencies increase, the total litter sizes at birth are increasing; however, the survival numbers at birth, 21 d, and weaning (about 45 d) show no differences between each other. The mortality observed is comparable to values reported by others, showing that the larger the litter size the greater the mortality. The effects of birth frequencies on litter weights and individual weights, analyzed by the analysis of variance (ANOVA), are shown in Table 5. The litter weights show a small positive correlation with higher birth frequencies (*P* > 0.05), while these data provide no positive or negative correlations between birth frequencies and the individual weights at birth, 21 d, and weaning.

### 3.4. Hematology and Blood Biochemistry

Hematology and blood biochemistry indices are important to animals for evaluation of their homeostasis and disease diagnosis. Tables 6 and 7 present the background data of hematology and blood biochemistry for outbred WZS minipigs aged from 2 to 12 months and this range of ages represents the main range encountered in toxicological and biological studies [2, 3, 20]. In 19 haematological and 12 blood biochemical parameters, only EO, BUN, CHOL, and TG show significant differences between male and female WZS minipigs. The main differences of haematological parameters between closed colony and inbred strain [21] focus on indices of hemoglobin and blood platelet, and there were 6 different indices of biochemical parameters.

### 3.5. Microsatellite Analysis

Twenty-six commonly used microsatellite loci were employed to analyze the genetic diversity of the outbred WZS minipigs by multi-PCR. Results of 44 randomly selected animals showed that the mean allelic numbers per locus were 8.37, the mean polymorphism information content (PIC) was 0.7069, and the mean heterozygosity (H) was 0.5478. The mean allelic numbers per locus, mean PIC, and mean H were significantly higher than those of inbred WZS minipigs reported elsewhere [12], which were 6.92, 0.6042, and 0.4478, respectively. Yao et al. had employed twenty-seven microsatellite markers recommended by the* Food and Agriculture Organization (FAO)* and the* International Society for Animal Genetics (ISAG)* to analyze the genetic diversities of WZS minipigs bred in original region, Hainan, China [22]. Their results are similar to our results. These data present that the outbred WZS minipigs are genetically stabilized breed with high heterozygosity and maintain the original characteristics.

### 3.6. Organ Coefficients

Seventy-four healthy WZS minipigs (43 males and 31 females) aged from 5 to 20 months were chosen to determine 6 organs weights and organ coefficients. Data are listed in Table 8. Student's* t*-test is employed to analyze the differences of organ weights and organ coefficients between males and females of the same age range. Only few organ weights of different aged animals show significant difference between males and females (*P* < 0.05); they are lung weight of animals aged 5–9 months and kidney weight of animals aged 10–15 months, but there was no significant difference for the organ coefficients (*P* > 0.05). Furthermore, the effects of age factor on organ weights and organ coefficients were analyzed by ANOVA. Results of ANOVA showed that organ weights increased significantly in accordance with the growths and increases of body weights (*P* < 0.0001); the organ coefficients of heart, liver, lung, and stomach decreased significantly with the growth of animals (*P* < 0.0001), but the decreases of spleen and kidney coefficients were not significant (*P* > 0.05).

### 3.7. Carcass Properties

Sun and Lu [23] had reported some pork characteristics of WZS minipigs based on 3 young and 3 adult minipigs [23]. In this study, we extensively determined the carcass properties of 11 adult WZS minipigs aged from 6 to 10 months. All detected carcass traits are shown in Tables 9 and 10. According to Table 9, there are no significant differences between males and females for all carcass properties. When compared to the same age range domestic pigs [24], all the traits of WZS minipigs are significantly lower, indicating a lean-type pig. For males, the comparisons between WZS minipigs and Tibet minipigs in China [24] show that skin thickness, back fat thickness, bone percentage, and skin percentage of WZS minipigs are higher than those of Tiber minipigs. Besides the above traits, the eye muscle area of WZS minipigs is also higher than that of Tibet minipigs for females. Results of sensory characters in Table 10 demonstrate that WZS minipigs are of perfect meat color, meat elasticity, and less marbling.

## 4. Conclusion

In conclusion, we report the detailed biological characteristics of outbred WZS minipigs cultivated for more than ten years. Series of background parameters have been detected out and proved to be stable in outbred WZS minipigs. Reproductive performances show that sexual maturity of sow is earlier than domestic pigs. Growth performances demonstrate that WZS minipigs have downsizing body shape and low body weight. Microsatellite analysis reveals the high heterozygosity of WZS minipigs. Organ coefficients, hematology, and blood biochemistry indices are stable. These results indicate that outbred WZS minipigs are suitable laboratory animals and could be widely used in comparative medicine and bioresearches in future.

## Figures and Tables

**Table 1:** Microsatellite loci and grouping in multi-PCR.

Group	Fluorescent labeling		
	FAM	HEX	TET
1	*SW*255	*SW*986, *SW*2519	*SW*R925, *SW*1118,* S*0018
2	*SW*1305, *SW*936	—	*SW*71, *SW*1035
3	*SW*511, *SW*790	*SW*1202, *SW*1408	*SW*2141, *SW*1129
4	*SW*943, *SW*65	*SW*520, *SW*2459	*SW*2001
5	*SW*268, *SW*903	*SW*486	*SW*2415, *SW*1553

**Table 2:** Growth performances of outbred WZS minipigs of different ages.

Age class	Sex	*n*	BW (kg)	HAW (cm)	BL (cm)	HD (cm)	HB (cm)	HG (cm)	AG (cm)	SG (cm)	BKL (cm)	BB (cm)	HL (cm)	FB (cm)	JW (cm)	CL (cm)	CG (cm)
Birth	Total	191	0.57 ± 0.11	11.65 ± 1.00	17.10 ± 1.25	5.83 ± 0.52	4.55 ± 0.48	17.67 ± 1.44	19.08 ± 1.86	4.16 ± 0.32	6.00 ± 0.58	3.86 ± 0.45	7.98 ± 0.55	4.28 ± 0.25	4.59 ± 0.25	5.50 ± 0.60	1.97 ± 0.16
	Male	96	0.59 ± 0.10	11.75 ± 0.95	17.22 ± 1.15	5.91 ± 0.49	4.63 ± 0.47	17.94 ± 1.26	19.51 ± 1.63	4.24 ± 0.29	6.04 ± 0.55	3.92 ± 0.43	8.11 ± 0.46	4.32 ± 0.27	4.65 ± 0.24	5.55 ± 0.57	1.99 ± 0.15
	Female	95	0.55 ± 0.11∗∗	11.54 ± 1.05	16.98 ± 1.34	5.76 ± 0.54	4.48 ± 0.48	17.39 ± 1.55∗	18.65 ± 1.99∗∗	4.09 ± 0.32∗∗	5.96 ± 0.61	3.80 ± 0.46	7.86 ± 0.60∗∗	4.24 ± 0.23∗	4.53 ± 0.24∗∗	5.46 ± 0.62	1.95 ± 0.16

1 month	Total	190	2.80 ± 0.69	18.27 ± 2.01	29.34 ± 2.62	9.47 ± 0.96	8.37 ± 1.03	30.19 ± 2.98	33.05 ± 3.37	5.82 ± 0.47	11.19 ± 1.28	7.23 ± 1.14	10.73 ± 0.98	5.71 ± 0.45	6.61 ± 0.54	7.82 ± 0.92	3.15 ± 0.30
	Male	95	2.76 ± 0.68	18.23 ± 1.94	29.27 ± 2.55	9.41 ± 1.00	8.26 ± 1.10	29.99 ± 3.10	32.64 ± 3.53	5.83 ± 0.49	11.17 ± 1.26	7.14 ± 1.19	10.74 ± 0.88	5.70 ± 0.49	6.61 ± 0.51	7.82 ± 0.83	3.17 ± 0.29
	Female	95	2.84 ± 0.70	18.31 ± 2.08	29.42 ± 2.70	9.53 ± 0.91	8.48 ± 0.94	30.38 ± 2.84	33.46 ± 3.16	5.82 ± 0.46	11.21 ± 1.31	7.32 ± 1.08	11.72 ± 1.07	5.72 ± 0.41	6.61 ± 0.57	7.81 ± 1.00	3.12 ± 0.32

2 months	Total	169	4.54 ± 1.66	22.30 ± 2.55	35.68 ± 4.29	11.15 ± 1.48	9.73 ± 1.44	34.81 ± 4.32	39.12 ± 5.45	6.54 ± 0.73	12.95 ± 1.71	8.52 ± 1.98	12.71 ± 1.02	6.31 ± 0.48	7.53 ± 0.72	9.82 ± 1.24	3.50 ± 0.38
	Male	91	4.43 ± 1.67	22.17 ± 2.43	35.65 ± 4.55	11.03 ± 1.51	9.67 ± 1.50	34.53 ± 4.35	38.66 ± 5.59	6.57 ± 0.76	13.02 ± 1.82	8.47 ± 2.11	12.70 ± 0.86	6.33 ± 0.46	7.57 ± 0.70	9.79 ± 1.24	3.45 ± 0.33
	Female	78	4.67 ± 1.65	22.44 ± 2.68	35.71 ± 3.98	11.29 ± 1.45	9.81 ± 1.38	35.13 ± 4.29	39.65 ± 5.28	6.50 ± 0.70	12.88 ± 1.59	8.58 ± 1.83	12.71 ± 1.19	6.29 ± 0.52	7.49 ± 0.73	9.87 ± 1.24	35.6 ± 0.43

3 months	Total	142	6.13 ± 1.92	25.70 ± 3.20	41.09 ± 4.69	12.64 ± 1.74	10.91 ± 1.45	39.28 ± 4.89	44.78 ± 5.72	7.08 ± 0.74	14.62 ± 2.04	9.22 ± 1.34	14.42 ± 1.27	6.90 ± 0.55	8.20 ± 0.65	11.46 ± 1.57	3.93 ± 0.47
	Male	71	6.02 ± 1.95	25.58 ± 3.17	41.05 ± 4.82	12.39 ± 1.57	10.85 ± 1.51	38.81 ± 4.67	44.58 ± 5.83	7.13 ± 0.78	14.61 ± 2.19	9.31 ± 1.35	14.47 ± 1.17	6.92 ± 0.58	8.16 ± 0.66	11.32 ± 1.53	3.88 ± 0.41
	Female	71	6.25 ± 1.90	25.83 ± 3.25	41.13 ± 4.60	12.89 ± 1.88	10.97 ± 1.39	39.75 ± 5.10	44.98 ± 5.65	7.02 ± 0.71	14.63 ± 1.89	9.13 ± 1.34	14.36 ± 1.36	6.89 ± 0.53	8.24 ± 0.65	11.59 ± 1.60	3.98 ± 0.52

4 months	Total	113	7.78 ± 2.17	28.07 ± 3.23	44.11 ± 4.43	14.21 ± 1.89	11.89 ± 1.38	43.12 ± 4.60	49.36 ± 6.74	7.41 ± 0.76	15.78 ± 1.87	10.20 ± 1.41	15.95 ± 0.99	7.23 ± 0.53	8.69 ± 0.68	12.88 ± 1.59	4.45 ± 0.57
	Male	59	7.72 ± 2.05	27.98 ± 3.09	45.12 ± 4.59	14.01 ± 1.72	11.77 ± 1.41	42.73 ± 4.56	48.92 ± 7.57	7.45 ± 0.79	15.57 ± 1.88	10.18 ± 1.50	15.86 ± 0.94	7.32 ± 0.54	8.80 ± 0.59	12.70 ± 1.55	4.32 ± 0.43
	Female	54	7.84 ± 2.32	28.16 ± 3.41	45.09 ± 4.44	14.43 ± 2.05	12.02 ± 1.36	43.56 ± 5.65	49.84 ± 5.72	7.37 ± 0.72	16.02 ± 1.86	10.22 ± 1.31	16.05 ± 1.04	7.15 ± 0.51	8.57 ± 0.75	13.07 ± 1.63	4.58 ± 0.67∗

5 months	Total	90	9.61 ± 2.37	30.33 ± 3.71	48.28 ± 4.57	15.43 ± 1.82	12.55 ± 1.47	46.40 ± 4.76	52.87 ± 6.93	8.05 ± 0.89	17.37 ± 2.15	10.99 ± 1.32	16.76 ± 1.11	7.56 ± 0.47	9.16 ± 0.73	13.69 ± 2.09	4.68 ± 0.60
	Male	50	9.23 ± 2.23	29.78 ± 3.05	48.13 ± 4.35	15.07 ± 1.74	12.37 ± 1.55	45.60 ± 4.58	52.33 ± 7.26	8.12 ± 1.00	16.86 ± 2.21	10.77 ± 1.31	16.59 ± 1.21	7.53 ± 0.46	9.11 ± 0.67	13.33 ± 1.96	4.54 ± 0.59
	Female	40	10.10 ± 2.47	31.00 ± 4.34	48.48 ± 4.87	15.89 ± 1.83∗	12.78 ± 1.35	47.40 ± 4.85	53.55 ± 6.52	7.98 ± 0.75	18.00 ± 1.90	11.25 ± 1.31	16.99 ± 0.93	7.59 ± 0.48	9.23 ± 0.80	14.15 ± 2.20	4.86 ± 0.56∗

6 months	Total	84	12.11 ± 2.99	32.64 ± 3.81	52.13 ± 5.43	16.80 ± 2.09	13.95 ± 1.61	50.64 ± 5.77	58.30 ± 5.88	8.51 ± 0.74	18.77 ± 2.29	11.95 ± 1.31	17.75 ± 1.47	7.93 ± 0.54	9.74 ± 0.90	14.90 ± 2.40	5.10 ± 0.68
	Male	48	11.26 ± 2.67	32.05 ± 3.41	51.71 ± 4.99	16.44 ± 1.80	13.69 ± 1.41	49.56 ± 5.16	56.99 ± 5.42	8.45 ± 0.71	18.52 ± 1.98	11.72 ± 1.27	17.42 ± 1.41	7.80 ± 0.50	9.62 ± 0.88	14.36 ± 2.27	4.94 ± 0.65
	Female	36	13.24 ± 3.06∗∗	33.43 ± 4.20	52.69 ± 5.98	17.28 ± 2.36	14.29 ± 1.81	52.07 ± 6.29	60.06 ± 6.09∗	8.60 ± 0.79	19.11 ± 2.64	12.25 ± 1.33	18.18 ± 1.45∗	8.10 ± 0.55∗	9.89 ± 0.91	15.63 ± 2.41∗	5.32 ± 0.67∗∗

7 months	Total	75	15.02 ± 3.50	34.89 ± 2.85	55.88 ± 3.92	18.33 ± 2.05	14.92 ± 1.49	54.54 ± 4.80	61.37 ± 6.19	9.08 ± 0.69	20.58 ± 1.90	12.75 ± 1.23	19.06 ± 1.37	8.37 ± 0.58	10.38 ± 0.85	16.30 ± 1.81	5.62 ± 0.64
	Male	37	14.49 ± 3.01	34.40 ± 2.67	55.93 ± 3.11	18.36 ± 1.69	14.78 ± 1.34	54.34 ± 4.23	60.35 ± 6.04	9.11 ± 0.66	20.71 ± 1.83	12.68 ± 1.15	18.71 ± 1.19	8.30 ± 0.54	10.17 ± 0.74	15.83 ± 1.72	5.38 ± 1.46
	Female	38	15.53 ± 3.80	35.37 ± 2.90	55.84 ± 4.40	18.30 ± 2.29	15.06 ± 1.60	54.73 ± 5.16	62.37 ± 6.00	9.07 ± 0.71	20.46 ± 2.00	12.81 ± 1.32	19.40 ± 1.43∗	8.43 ± 0.62	10.58 ± 0.89∗	16.76 ± 1.74∗	5.86 ± 0.67

8 months	Total	67	16.95 ± 4.16	36.52 ± 4.75	57.88 ± 4.72	18.88 ± 2.56	15.65 ± 1.64	57.06 ± 6.71	64.06 ± 7.41	9.43 ± 0.69	20.90 ± 2.27	13.51 ± 1.45	20.19 ± 1.64	8.71 ± 0.68	10.96 ± 0.96	16.86 ± 2.36	5.85 ± 0.67
	Male	37	16.92 ± 0.65	36.56 ± 4.96	57.81 ± 4.28	18.93 ± 2.60	15.83 ± 1.66	57.27 ± 6.59	64.68 ± 6.95	9.56 ± 0.62	21.21 ± 2.29	13.70 ± 1.45	19.96 ± 1.27	8.76 ± 0.65	10.84 ± 0.86	16.83 ± 2.21	5.66 ± 0.56
	Female	30	16.97 ± 1.31	36.46 ± 4.66	57.97 ± 5.35	18.82 ± 2.59	15.45 ± 1.68	56.80 ± 7.07	63.31 ± 8.18	9.28 ± 0.80	20.51 ± 2.30	13.29 ± 1.48	20.47 ± 1.95	8.64 ± 0.74	11.10 ± 1.03	16.91 ± 2.54	6.07 ± 0.70∗

9 months	Total	62	18.74 ± 4.62	37.73 ± 4.30	59.54 ± 5.20	19.83 ± 2.56	15.71 ± 1.67	58.94 ± 6.72	65.92 ± 7.03	9.64 ± 0.86	21.81 ± 2.29	13.65 ± 1.51	20.70 ± 1.66	8.80 ± 0.75	11.12 ± 1.11	17.54 ± 2.22	6.01 ± 0.76
	Male	37	19.19 ± 4.56	37.81 ± 4.27	59.39 ± 4.94	19.99 ± 2.50	15.86 ± 1.70	59.46 ± 6.66	66.21 ± 7.03	9.83 ± 0.87	21.92 ± 2.37	13.71 ± 1.52	20.69 ± 1.65	8.96 ± 0.79	11.06 ± 1.18	17.48 ± 2.22	5.91 ± 0.64
	Female	25	18.09 ± 4.81	37.62 ± 4.31	59.76 ± 5.42	19.61 ± 2.70	15.49 ± 1.68	58.16 ± 6.97	65.48 ± 7.00	9.36 ± 0.82	21.66 ± 2.20	13.55 ± 1.54	20.71 ± 1.73	8.57 ± 0.63∗	11.22 ± 1.05	17.62 ± 2.17	6.16 ± 0.86

10 months	Total	54	21.96 ± 5.69	39.66 ± 4.14	63.02 ± 5.89	21.62 ± 2.71	17.01 ± 1.64	63.07 ± 7.09	71.17 ± 7.66	10.11 ± 0.68	23.01 ± 2.12	14.77 ± 1.49	21.95 ± 1.63	9.08 ± 1.05	12.02 ± 1.05	17.95 ± 1.83	6.38 ± 0.77
	Male	33	22.14 ± 5.15	39.46 ± 4.46	63.58 ± 5.99	21.64 ± 2.40	16.85 ± 1.49	63.09 ± 5.81	71.31 ± 6.69	10.16 ± 0.65	22.69 ± 2.05	14.70 ± 1.24	21.74 ± 1.34	8.97 ± 1.08	12.01 ± 0.98	17.77 ± 1.82	6.27 ± 0.54
	Female	21	21.69 ± 6.47	39.98 ± 3.43	62.15 ± 5.51	21.59 ± 3.10	17.26 ± 1.83	63.05 ± 8.70	70.95 ± 8.83	10.01 ± 0.76	23.50 ± 2.19	14.86 ± 1.86	22.29 ± 2.00	9.25 ± 0.87	12.03 ± 1.18	18.22 ± 1.66	6.55 ± 0.85

11 months	Total	50	23.36 ± 6.44	40.93 ± 3.55	64.78 ± 5.32	21.77 ± 2.62	17.37 ± 1.81	65.02 ± 7.50	71.30 ± 8.00	10.32 ± 0.80	23.69 ± 1.93	14.99 ± 1.56	22.41 ± 1.27	9.33 ± 0.72	12.24 ± 1.04	18.38 ± 1.97	6.51 ± 0.74
	Male	30	23.65 ± 6.74	41.06 ± 4.13	64.66 ± 5.61	21.92 ± 2.58	17.37 ± 1.68	65.57 ± 7.25	71.54 ± 7.65	10.43 ± 0.76	24.10 ± 1.86	15.03 ± 1.48	22.52 ± 1.22	9.44 ± 0.65	12.34 ± 1.04	18.52 ± 2.07	6.42 ± 0.62
	Female	20	22.94 ± 6.27	40.67 ± 2.55	64.95 ± 4.83	21.53 ± 2.74	17.37 ± 2.05	64.20 ± 8.15	70.95 ± 8.73	10.16 ± 0.89	23.08 ± 2.04	14.93 ± 1.72	22.24 ± 1.39	9.15 ± 0.85	12.09 ± 1.07	18.18 ± 1.78	6.63 ± 0.84

12 months	Total	39	26.21 ± 6.24	42.96 ± 3.37	67.38 ± 6.35	22.94 ± 2.82	17.59 ± 2.74	67.49 ± 7.74	74.69 ± 9.19	10.63 ± 0.78	24.75 ± 2.28	15.86 ± 1.64	23.19 ± 1.58	9.68 ± 0.70	12.70 ± 1.17	19.13 ± 2.11	6.72 ± 0.72
	Male	25	27.05 ± 5.76	42.92 ± 3.48	67.80 ± 5.78	23.28 ± 2.40	18.01 ± 1.78	67.88 ± 6.51	75.10 ± 8.30	10.86 ± 0.72	24.56 ± 1.92	15.88 ± 1.26	23.19 ± 1.36	9.90 ± 0.55	12.92 ± 1.20	19.28 ± 2.20	6.78 ± 0.63
	Female	14	24.72 ± 7.35	43.04 ± 3.32	66.64 ± 7.22	22.38 ± 3.54	16.87 ± 3.92	66.79 ± 9.38	74.00 ± 10.71	10.22 ± 0.81∗	25.07 ± 2.83	15.83 ± 2.17	23.19 ± 1.99	9.36 ± 0.85∗∗	12.33 ± 0.99	18.14 ± 4.12	6.51 ± 0.78

Note: comparisons between the males and females of the same age class. ∗*P* < 0.05and ∗∗*P* < 0.01.

**Table 3:** Parameters of reproductive behavior of outbred WZS minipigs.

*n*	Puberty (d)	Estrus cycle (d)	Behavioral estrus (d)	Gestation (d)
45	93.65 ± 3.50	21.16 ± 0.75	4.18 ± 0.31	113.0 ± 2.33

**Table 4:** Litter sizes of outbred WZS minipigs at birth, 21 d, and weaning in different birth frequencies.

Birth frequency	Litter size				ANOVA, *P*
	Total	At birth	At 21 d	At weaning	
First birth (*n* = 40)	6.35 ± 1.53	6.23 ± 1.53	5.63 ± 1.92	5.45 ± 1.95	0.1288
Second birth (*n* = 26)	7.08 ± 1.75	6.85 ± 2.07	6.57 ± 1.99	6.43 ± 1.94	0.2963
Multibirth (*n* = 30)	7.47 ± 1.96	6.87 ± 1.78	6.64 ± 1.87	6.12 ± 1.79	0.0635
ANOVA, *P*	0.0281	0.2337	0.1602	0.4283	—

**Table 5:** Litter weights and body weights of outbred WZS minipigs in different frequencies.

Birth frequency	Litter weight (kg)	Individual weight (kg)		
		At birth	At 21 d	At weaning
First birth (*n* = 40)	3.54 ± 1.07	0.57 ± 0.14	2.24 ± 0.67	3.35 ± 1.23
Second birth (*n* = 26)	3.71 ± 1.25	0.56 ± 0.13	2.30 ± 0.81	3.37 ± 1.29
Multibirth (*n* = 30)	4.13 ± 0.90	0.56 ± 0.12	2.28 ± 0.64	3.18 ± 0.92
ANOVA, *P*	0.7302	0.7856	0.7119	0.3244

**Table 6:** Determination of haematological parameters.

Parameters	Abbreviation	Unit	Male (*n* = 39)	Female (*n* = 45)
White blood cells	WBC	1 × 10^9^/L	17.47 ± 4.50	16.59 ± 6.19
Red blood cells	RBC	1 × 10^12^/L	8.58 ± 2.48	8.87 ± 1.25
Haemoglobin	HB	g/L	155.37 ± 38.90	156.44 ± 18.66
Hematocrit	HCT	%	51.76 ± 12.70	51.81 ± 6.57
Mean cell volume	MCV	fL	66.10 ± 5.84	58.80 ± 5.83
Mean cell hemoglobin	MCH	Pg	18.32 ± 1.78	17.76 ± 1.62
Mean cell hemoglobin concentration	MCHC	g/L	300.26 ± 13.06	302.44 ± 11.74
Total platelet count	PLT	1 × 10^9^/L	308.47 ± 121.70	363.96 ± 95.69
Platelet distribution width	PDW	fL	13.81 ± 3.04	14.48 ± 1.71
Platelet distribution width-SD	RDW-SD	fL	42.63 ± 3.77	42.46 ± 4.57
Platelet distribution width-CV	RDW-CV	%	21.65 ± 3.07	22.23 ± 2.39
Mean platelet volume	MPV	fL	10.35 ± 1.19	10.90 ± 0.78
Platelet-large cell ratio	P-LCR	%	31.31 ± 8.94	33.71 ± 5.27
Plateletcrit	PCT	%	0.21 ± 0.06	0.21 ± 0.05
Neutrophils	NEUTRO	1 × 10^9^/L	5.96 ± 2.53	6.96 ± 2.68
Lymphocytes	LYMPHO	1 × 10^9^/L	10.20 ± 4.39	9.61 ± 4.98
Monocytes	MONO	1 × 10^9^/L	0.60 ± 0.29	0.60 ± 0.22
Eosinophils	EO	1 × 10^9^/L	0.47 ± 0.23	0.36 ± 0.15∗
Basophils	BASO	1 × 10^9^/L	0.24 ± 0.19	0.21 ± 0.10

Note: results of *t-*test and comparison between males and females. ∗*P* < 0.05.

**Table 7:** Determination of the haemal biochemical parameters.

Parameters	Abbreviation	Unit	Male (*n* = 35)	Female (*n* = 42)
Alanine aminotransferase	ALT	U/L	88.33 ± 24.27	79.26 ± 16.93
Aspartate aminotransferase	AST	U/L	63.00 ± 26.03	61.78 ± 32.28
Alkaline phosphatase	ALP	U/L	239.07 ± 135.90	273.13 ± 146.09
Total protein	TP	g/L	85.25 ± 5.29	82.87 ± 10.79
Albumin	ALB	g/L	35.41 ± 3.24	32.89 ± 6.16
Globulin	GLB	g/L	49.84 ± 5.81	49.99 ± 8.67
Blood urea nitrogen	BUN	mmol/L	6.47 ± 5.48	4.15 ± 1.49∗
Creatinine	CREA	*μ*mol/L	148.86 ± 62.16	127.91 ± 37.18
Glucose	GLU	mmol/L	5.81 ± 2.95	5.65 ± 2.02
Cholesterol	CHOL	mmol/L	1.93 ± 0.55	2.69 ± 0.58∗
Total bilirubin	TBILI	*μ*mol/L	0.50 ± 0.37	0.43 ± 0.69
Triglycerides	TG	mmol/L	0.45 ± 0.19	0.59 ± 0.24∗

Note: results of *t-*test and comparison between males and females. ∗*P* < 0.05.

**Table 8:** Organ absolute weight and organ coefficients (group mean values) of outbred WZS minipigs.

Parameter	Aged 5–9 months			Aged 10–15 months			Aged 16–20 months			ANOVA of total values, *P*
	Total	♂ (*n* = 17)	♀ (*n* = 17)	Total	♂ (*n* = 16)	♀ (*n* = 5)	Total	♂ (*n* = 7)	♀ (*n* = 6)	
Age, month	8.24 ± 0.34	8.19 ± 0.25	8.31 ± 0.43	13.55 ± 1.06	13.78 ± 1.09	12.80 ± 0.45	17.96 ± 1.84	18.93 ± 1.13	16.83 ± 1.94	—
Body weight (kg)	20.33 ± 5.44	19.17 ± 5.06	21.28 ± 5.75	31.98 ± 6.95	31.51 ± 6.00	33.48 ± 10.13∗	36.73 ± 7.13	35.64 ± 5.76	38.00 ± 8.87	<0.0001
Heart										
Weight (g)	92.36 ± 20.89	91.29 ± 21.19	93.31 ± 21.23	111.09 ± 17.36	106.92 ± 15.06	126.73 ± 18.37	117.86 ± 24.52	109.56 ± 16.95	127.55 ± 29.81	<0.0001
Coefficients (%)	0.45 ± 0.14	0.48 ± 0.13	0.45 ± 0.09	0.35 ± 0.050	0.35 ± 0.06	0.35 ± 0.02	0.32 ± 0.036	0.31 ± 0.03	0.34 ± 0.04	<0.0001
Lung										
Weight (g)	174.91 ± 56.39	153.86 ± 61.85	194.72 ± 43.74∗	223.18 ± 44.25	219.70 ± 47.99	237.10 ± 23.79	221.24 ± 60.89	223.16 ± 43.23	219.00 ± 81.51	<0.0001
Coefficients (%)	0.89 ± 0.34	0.79 ± 0.19	0.99 ± 0.42	0.70 ± 0.16	0.71 ± 0.16	0.66 ± 0.16	0.60 ± 0.12	0.63 ± 0.10	0.57 ± 0.13	<0.0001
Liver										
Weight (g)	359.31 ± 81.32	339.50 ± 83.02	377.95 ± 77.54	454.46 ± 88.28	439.42 ± 89.23	507.10 ± 70.08	483.50 ± 90.63	489.73 ± 85.62	476.23 ± 103.90	<0.0001
Coefficients (%)	1.81 ± 0.29	1.78 ± 0.19	1.84 ± 0.36	1.42 ± 0.21	1.42 ± 0.23	1.39 ± 0.14	1.33 ± 0.19	1.38 ± 0.16	1.27 ± 0.23	0.0002
Spleen										
Weight (g)	36.37 ± 8.34	35.14 ± 8.18	37.46 ± 8.57	52.91 ± 13.18	51.46 ± 13.85	57.98 ± 10.45	58.96 ± 17.07	58.54 ± 15.15	59.46 ± 20.57	<0.0001
Coefficients (%)	0.19 ± 0.048	0.19 ± 0.04	0.18 ± 0.06	0.17 ± 0.044	0.17 ± 0.05	0.16 ± 0.02	0.16 ± 0.049	0.16 ± 0.02	0.16 ± 0.07	0.2443
Kidney (L)										
Weight (g)	39.76 ± 10.38	36.19 ± 9.71	43.10 ± 10.14	60.30 ± 14.78	56.04 ± 13.72	73.08 ± 10.31∗	61.51 ± 15.21	64.91 ± 16.61	57.55 ± 13.74	<0.0001
Coefficients (%)	0.20 ± 0.069	0.19 ± 0.03	0.20 ± 0.07	0.19 ± 0.060	0.18 ± 0.04	0.22 ± 0.09	0.17 ± 0.033	0.18 ± 0.04	0.16 ± 0.02	0.2793
Kidney (R)										
Weight (g)	40.64 ± 11.09	36.69 ± 10.29	44.34 ± 10.81	60.73 ± 13.47	56.98 ± 13.05	71.96 ± 7.43∗	62.15 ± 14.03	63.02 ± 17.45	61.12 ± 10.25	<0.0001
Coefficients (%)	0.20 ± 0.074	0.19 ± 0.03	0.21 ± 0.07	0.19 ± 0.057	0.18 ± 0.04	0.21 ± 0.09	0.17 ± 0.035	0.18 ± 0.04	0.16 ± 0.03	0.2212
Stomach										
Weight (g)	190.76 ± 34.04	194.52 ± 38.44	186.65 ± 29.81	245.42 ± 46.42	241.94 ± 48.83	256.73 ± 41.66	258.42 ± 65.05	234.47 ± 43.29	286.35 ± 78.53	<0.0001
Coefficients (%)	0.94 ± 0.28	1.01 ± 0.29	0.88 ± 0.27	0.77 ± 0.18	0.77 ± 0.20	0.77 ± 0.09	0.70 ± 0.12	0.66 ± 0.05	0.75 ± 0.17	<0.0001

Note: results of *t*-test and comparisons between males and females of the same age ranges. ∗*P* < 0.05.

**Table 9:** Determination of carcass properties in outbred WZS minipigs.

Item	Means		
	Total (*n* = 11)	Male (*n* = 5)	Female (*n* = 6)
Body weight (kg)	24.55 ± 7.28	22.92 ± 10.21	25.92 ± 4.20
Slaughter weight (kg)	16.16 ± 4.87	14.89 ± 6.65	17.23 ± 3.02
Blood weight (kg)	1.01 ± 0.17	0.99 ± 0.09	1.03 ± 0.21
Head weight (kg)	2.97 ± 0.73	2.44 ± 0.82	3.33 ± 0.41
Forepaw weight (kg)	0.31 ± 0.09	0.29 ± 0.14	0.33 ± 0.07
Rear foot weight (kg)	0.38 ± 0.11	0.33 ± 0.13	0.43 ± 0.08
Suet (including kidney) (kg)	0.31 ± 0.03	0.30 ± 0.02	0.31 ± 0.04
Lean meat weight (kg)	8.57 ± 2.37	6.97 ± 2.70	9.84 ± 1.06
Fat weight (kg)	2.50 ± 1.72	2.64 ± 2.55	2.39 ± 1.01
Bone weight (kg)	1.95 ± 0.54	1.57 ± 0.60	2.25 ± 1.01
Skin weight (kg)	2.22 ± 0.79	1.94 ± 1.16	2.45 ± 0.34
Skin thickness (mm)	3.64 ± 0.92	3.40 ± 1.14	3.83 ± 0.75
Back fat thickness (mm)	11.70 ± 6.05	11.47 ± 6.76	11.90 ± 4.97
Carcass length (cm)	47.30 ± 4.72	43.50 ± 3.11	49.83 ± 3.87
Eye muscle area (cm^2^)	15.55 ± 2.81	14.66 ± 2.66	16.29 ± 1.87
Dressing percentage (%)	66.03 ± 2.52	65.55 ± 3.67	66.35 ± 1.95
Lean meat percentage (%)	57.11 ± 4.86	55.61 ± 6.57	58.31 ± 3.30
Fat meat percentage (%)	15.31 ± 6.35	17.17 ± 8.46	13.82 ± 4.56
Bone percentage (%)	12.64 ± 2.58	12.63 ± 3.87	12.64 ± 1.44
Skin percentage (%)	14.36 ± 1.45	14.19 ± 1.97	14.49 ± 1.11

Note: no significant differences were found between males and females by *t*-test.

**Table 10:** Results of sensory character (*n* = 11).

Item	Meat color^a^		Elasticity^b^		Marbling^c^	
	Fresh meat	Cool meat	Fresh meat	Cool meat	Fresh meat	Cool meat
Result	2.57 ± 0.62	2.76 ± 1.41	Good	Good	—	4.35 ± 0.19

Note: ^a^meat sensory character was evaluated as described previously [11]. Meat color categories: 1, pale-purplish gray; 2, grayish pink; 3, reddish pink; 4, purplish red; 5, dark, purplish red. ^b^Meat elasticity was classified to three levels: good, normal, and poor by hand pressing. ^c^For meat marbling, score of 1 is devoid of fat and 10 has abundant marbling.
